# Role of leptin receptor gene polymorphisms in susceptibility to the development of essential hypertension: a case–control association study in a Northern Han Chinese population

**DOI:** 10.1038/jhh.2013.149

**Published:** 2014-02-13

**Authors:** Y Liu, Y-Q Lou, K Liu, J-L Liu, Z-G Wang, J Wen, Q Zhao, S-J Wen, L Xiao

**Affiliations:** 1 Department of Hypertension Research, Beijing Anzhen Hospital, Capital Medical University and Beijing Institute of Heart Lung and Blood Vessel Diseases, Beijing, PR China; 2 Department of Pulmonary Disease, Shanghai Chest Hospital, Shanghai Jiaotong University, Shanghai, PR China; 3 Emergency Department, China MeiTan General Hospital, National Mining Medical Center, Beijing, PR China; 4 Department of Medicine, Division of Cardiology, Northwestern University, Chicago, IL, USA; 5 Department of Medicine & Center for Cardiovascular Research, University of Illinois at Chicago, Chicago, IL, USA

**Keywords:** leptin receptor, polymorphism, essential hypertension, Chinese, linkage disequilibrium, haplotypes

## Abstract

In order to explore the potential association between the leptin receptor (LEPR) gene polymorphisms and essential hypertension (EH) risk in the Northern Han Chinese population, we recruited 823 hypertensive subjects and 491 healthy control subjects from the Northern Han Chinese. Genotyping was performed to identify the Lys109Arg, Gln223Arg and Lys656Asn polymorphisms of the *LEPR* gene. Significant associations were found in a dominant genetic model ([GG+AG] vs AA), *P*=0.007, odds ratio (OR)=3.697, 95% confidence interval (CI) 1.442–9.482), and in homozygote comparison (GG vs AA, *P*=0.005, OR=3.890, 95% CI 1.501–10.077) for the Gln223Arg polymorphism. No significant association could be found between Lys109Arg or Lys656Asn polymorphism and EH risk. Linkage disequilibrium was detected between the Lys109Arg and Gln223Arg polymorphisms, and haplotype analyses identified that the G-A haplotype was a protective haplotype for EH. Our studies demonstrated that the *LEPR* Gln223Arg polymorphism had an important role in a patient's susceptibility to EH in the Northern Han Chinese population.

## Introduction

Essential hypertension (EH) is a worldwide escalating health problem, and 27.2% of the adult Chinese population from age 35 to 74 years old suffered from this disease.^1^ EH represents a major risk factor for stroke, myocardial infarction and renal disease, and often occurs in combination with other metabolic complications, such as hyperlipidemia, obesity and insulin resistance. The pathogenesis of EH is complex, characterized by the involvement of multiple genes and environmental factors. Leptin and leptin receptor (LEPR) genes have been suggested to be candidate genes for hypertension via their direct effects on regulation of blood pressure (BP) and adipose tissue metabolism, or indirect effect on obesity.^2^

Leptin is an adipocyte-derived hormone that suppresses food intake and increases energy expenditure by binding to and activating its specific receptor in the hypothalamus.^3, 4^ In addition, it may also affect BP by stimulating sympathetic outflow.^5, 6^ Leptin exerts its effects through the transmembrane LEPR, the gene that is located on the human chromosome 1P32. The LEPR, which has several isoforms, is a single-transmembrane protein belonging to the cytokine receptor family. A sequence variation in the *LEPR* gene will impair the efficacy of leptin binding to its receptor,^7^ attenuating the favorably regulatory effects on BP. Meanwhile, polymorphisms in the *LEPR* gene have been reported being associated with high-plasma leptin level, indicating leptin resistance and a lower whole-body plasma norepinephrine spillover, which is an index of blunted sympathetic nerve activity.^8^

**Figure 1:**
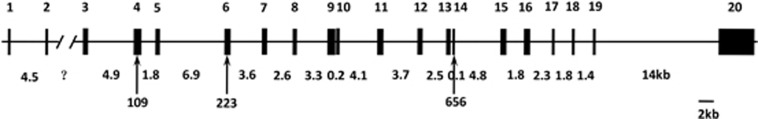
Polymorphism sites of the human *LEPR* gene. The human *LEPR* gene is composed of 20 exons and spans >70 kb of DNA. The exons are represented as vertical bars at double scale and are numbered above each exon. Estimated intron sizes are shown below except for intron no. 2 whose size is unknown. Polymorphisms are shown below the map and are identified by their amino-acid number.

Several previous studies had investigated the relationship between the polymorphisms of *LEPR* gene (Lys109Arg, Gln223Arg and Lys656Asn) and EH risk in different ethnic groups, but the results are controversial. Wiedemann *et al.*^9^ failed to identify any association in the German population, whereas the study by Rosmond *et al.*^10^ suggested that the variants of Lys109Arg and Gln223Arg seem to protect from hypertension development in the Swedish population. In order to clarify the roles of these three polymorphisms of the *LEPR* gene in the development of hypertension, we conducted a case-controlled study in the Northern Han Chinese population. Genomic locations and related mapping data were obtained from the National Center for Biotechnology Information (Figure 1).

## Materials and Methods

### Study population

All normotensive participants and hypertensive patients were screened at the physical examination center and hypertension clinic at the Beijing Anzhen Hospital, Capital Medical University, Beijing, China. A total of 491 healthy, normotensive subjects (NT group) and 823 hypertensive patients (EH group) were screened. BP was accurately measured three times with a mercury sphygmomanometer by experienced internists at their offices. Measurements were recorded after the subjects had been seated in a chair with their feet on the floor and their arms supported at heart level for 10 min. The definition of normotension (systolic blood pressure (SBP)<120 mm Hg and diastolic blood pressure (DBP)<80 mm Hg) and hypertension (SBP⩾140 mm Hg or DBP⩾90 mm Hg) were based on the BP classification of the seventh report of the Joint National Committee on Prevention, Detection, Evaluation and Treatment of High Blood Pressure (JNC-VII).^11^ All hypertensive patients were diagnosed as being EH and without any treatment of antihypertensive medications. No EH patient is concurrently diagnosed with any other known disease, including secondary hypertension, primary renal disease, diabetic mellitus, hepatic disorders, cancer or other endocrine diseases, for example, hyperthyroidism and so on. Smokers were defined as cigarette consumers who had smoked ⩾100 cigarettes; and drinkers were defined as alcohol consumers who drank ⩾12 times during the past year.^12, 13^ Obese was defined as body mass index ⩾25 kg m^−2^ according to the World Health Organization obesity guidelines on Asians.^14^ This study complied with the Declaration of Helsinki. All participants signed a consent form, and the study was approved by the Anzhen Hospital Ethics Committee of the Capital Medical University.^15^

### Genotyping

Patient's peripheral blood sample was taken into EDTA-containing receptacles. Genomic DNA was extracted from the peripheral blood according to standard phenol–chloroform methods. We genotyped single-nucleotide polymorphisms (SNPs) using the TaqMan assay. The *LEPR* SNP TaqMan probes and primers were obtained from Applied Biosystems Assay-by-Design Service for SNP genotyping (Foster City, CA, USA). The sample DNA was amplified by PCR on a GeneAmp PCR System 9700 thermal cycler (Applied Biosystems) following the manufacturer's recommendations. Genotypes were differentiated by analyzing the fluorescence levels of PCR products using an ABI PRISM 7900HT Sequence Detector (Applied Biosystems). Genotyping was performed blindly to all other data.

### Statistics

In our experimental design, we used the published data in the literature to calculate the sample size, and the calculated power value was >0.8. We first used Gln223Arg data as a sample group to calculate the power value. We then used the statistical software ‘Power and Sample Size Calculations'^16^ to calculate the power value that was 0.882.

We used SPSS (Version 17.0; SPSS, Chicago, IL, USA) for database management and statistical analysis. All comparisons between specific groups for continuous variables were made using a two-sample *t*-test. Allelic and genotypic frequencies were compared between the hypertensive cases and the normotensive controls by using the *χ*^2^-test. To test for an association between each SNP and hypertension risk, we computed the overall genotypic test of association and genetic models (dominant, recessive, additive, allele and homozygote). A multinomial logistic regression was used to study the effect of *LEPR* Lys109Arg, Gln223Arg and Lys656Asn variants on hypertension status to allow incorporation of other variables into the model. All tests of association were adjusted for age, gender, body mass index, total cholesterol, high-density lipoprotein cholesterol, low-density lipoprotein cholesterol, serum triglyceride and plasma glucose levels, as well as smoking and drinking habits. All analyses used the two-tailed estimation of significance. The statistical significance was defined by *P*<0.05. Multiple testing was adjusted using the Bonferroni correction.

The presence of Hardy–Weinberg equilibrium was tested by the *χ*^2^-test for goodness of fit based on a web program (http://ihg.gsf.de/cgi-bin/hw/hwa1.pl).

Construction of the linkage disequilibrium map and haplotype blocks within polymorphisms of the *LEPR* gene was based on genotypes and utilized Haploview software (version 4.1) (http://www.broad.mit.edu/mpg/haploview/).^17^ The expectation maximization algorithm^18^ was performed to estimate haplotype frequencies and to obtain the best haplotype configuration for each multi-locus genotype. All haplotypes with frequency >1% in the combined case and control samples were examined. The *χ*^2^-test was conducted to compare the haplotype distributions between the hypertensives and the normotensives. Haplotype-specific testing was performed to compare a specific haplotype with the others. Assuming the highly prevalent haplotype as the baseline, each of the other haplotype was then compared with the baseline haplotype using a logistic regression model.^14^

## Results

### Characteristics of the participants

A total of 1314 unrelated participating subjects comprising 823 hypertensive patients (531 men and 292 women; mean age 51.40±9.38) and 491 normotensive control subjects (293 men and 198 women; mean age 50.47±7.91) were recruited for the present study. The clinical and laboratory parameters of cases and controls were summarized in Table 1. In addition to BP changes, significant differences in body mass index, triglyceride level, glucose level and the ratio of drinkers were also observed between the EH and NT groups.

### Association analyses

Among all the participants, 99.3% samples of Lys109Arg polymorphism, 98.8% samples of Gln223Arg polymorphism and 96% samples of Lys656Asn polymorphism were successfully detected in the laboratory. No deviation from the Hardy–Weinberg expectation was observed for the effect of *LEPR* Lys109Arg, Gln223Arg or Lys656Asn variants in either the EH group or the NT group (data not shown). The genotype frequencies for the three polymorphisms in the *LEPR* gene are shown in Table 2. Chi-square analyses indicated that the Gln223Arg polymorphism was significantly associated with EH. In contrast, no significant difference was found for the Lys109Arg or Lys656Asn polymorphisms. Our stratification analysis result did not show difference in *LEPR* polymorphisms (Lys109Arg, Gln223Arg and Lys656Asn) on triglyceride levels (data not shown) or obesity (Table 3).

The data were then subjected to logistic regression analysis after adjusting for confounding risk variables. Significant association of Gln223Arg polymorphism with EH risk was found in the dominant genetic model ([GG+AG] vs AA), *P*=0.007, odds ratio (OR)=3.697, 95% confidence interval (CI) 1.442–9.482), and in homozygote comparison (GG vs AA, *P*=0.005, OR=3.890, 95% CI 1.501–10.077). For Lys656Asn polymorphism, a significantly higher prevalence of C allelic frequencies (*P*=0.044, OR=1.460, 95% CI 1.011–2.108) in the hypertensives than the normotensives was observed, suggesting that the C allele could be a risk factor for hypertension in the Northern Han Chinese. In addition, significant associations were found in the dominant genetic model (CC+GC) vs GG, *P*=0.034, OR=1.515, 95% CI 1.032–2.225 and additive genetic model (CC vs GC vs GG, *P*=0.041, OR=1.479, 95% CI 1.017–2.152). No significant association could be found between Lys109Arg polymorphism and EH risk (Table 4). To correct type I error, we applied the Bonferroni correction on the criteria of *P*=0.05/3 because three polymorphisms were tested. The statistical significance of the dominant genetic model and homozygote comparison between Gln223Arg polymorphism and EH risk remained, but the significance of allele comparison, dominant genetic model and additive genetic model between Lys656Asn polymorphism and EH risk disappeared.

### Haplotype analyses

The Lys109Arg and Gln223Arg variants were in linkage disequilibrium (*D*′=0.83, *r*^2^=0.48). The Haploview program revealed that the Lys109Arg and Gln223Arg polymorphisms are in the same linkage disequilibrium block. The haplotype analyses of the two polymorphisms of the *LEPR* gene in hypertension and control subjects are shown in Table 5. The G-A haplotype was a protective haplotype (*P*=0.012, OR=0.482, 95% CI 0.273–0.850), whereas the G-G, A-A and A-G haplotypes were not associated with EH in the Northern Han Chinese (*P*=0.132, 0.647 and 0.660, respectively).

The relative effects of these haplotypes were evaluated by the logistic regression. As the most highly prevalent haplotype, the G-G haplotype was defined as the baseline haplotype. Compared with the baseline haplotype, the G-A haplotype showed to be associated with a protective haplotype to EH (*P*=0.010, OR=0.474, 95% CI 0.269–0.837).

## Discussion

Both genetic and environmental factors have important roles in the pathogenesis and progression of EH.^14, 19^ Genetic factor was considered to be of clinical importance by physicians and researchers in the pathogenesis, diagnosis, treatment and prevention of hypertension. With the advent of the human genome project and the international HapMap project, SNPs have become increasingly prominent in the studies of both multifactorial and multi-genomic diseases.^15^ The aim of the present study was to determine whether the polymorphisms in the *LEPR* gene were associated with EH development in a Northern Han Chinese population and whether there was a SNP–SNP interaction in the *LEPR* gene.

In our studies, no difference in genotype distribution of Lys109Arg between the EH patients and the controls was found, which is consistent with a previous report by Gu *et al.*^20^ However, our studies identified that the Gln223Arg polymorphism of the *LEPR* gene was associated with EH in the Northern Han Chinese, and G allele carriers of Gln223Arg (GG+AG) showed higher risks of hypertension than AA homozygotes (*P*=0.007, OR=3.697, 95% CI 1.442–9.482). Interestingly, the study by Gu *et al.*^20^ showed a different result that Gln223Arg (AA+AG) had higher risk of hypertension than GG carriers (*P*=0.035, OR=1.549, 95% CI 1.031–2.036). It is likely that this discrepancy might be attributed to the following three reasons. First, in the report by Gu *et al.*,^20^ subjects were selected exclusively from the metropolitan areas of Shanghai and Nanjing, which are two cities in southern China with majority residential population of southern Han Chinese; whereas northern Chinese population was intentionally selected in our study in the northern areas of China. As China is the most populous country in the world, and the largest Chinese ethnic group Han Chinese (>90% of the total Chinese population) is further classified into two subgroups, northern and southern Han Chinese, with reportedly distinct genetic backgrounds.^21, 22^ Second, two different inclusion criteria were used to determine the BP levels of the control group. The criteria for normotensive controls in our study were: SBP<120 mm Hg and DBP<80 mm Hg; whereas the inclusion criteria in the report by Gu *et al.*^20^ were: SBP<130 mm Hg and DBP<85 mm Hg. The different inclusion criteria would influence the results, as BP ranging from 120 to 139 mm Hg SBP and/or 80 to 89 mm Hg DBP is defined as prehypertension. People with prehypertension are considered to be at high risk of developing hypertension.^11^ Therefore, we believe that our inclusion criteria were stricter than the other report. Third, the adjustment variables, for example, smoking and drinking habits were considered in our analysis, but not in the other report by Gu *et al.*^20^ It has been suggested that smoking and alcohol intake are causative factors in the development of hypertension,^23, 24^ thus they should be taken into account in our studies.

We did not find significant association between Lys656Asn polymorphism and EH risk. A previous report by de Luis Roman *et al.*^25^ showed that SBP decreased significantly in the Lys656 (GG) homozygotes, but not in the carriers of the Asn656 (C) allele. The ethnic background difference among these populations thus could be a main reason for such a variation. Furthermore, EH is a complex polygenic disease responsive to multiple environmental factors. As multiple genes and genetic interactions have been implicated in the regulation of BP, a single polymorphism of one gene likely has less impact on an individual's phenotype. Moreover, other causative factors for hypertension including environmental factors and lifestyle habits (for example, salt intake, smoking and alcohol consumption) ought to be considered in studies of EH.^19^ Currently, the relationship between Lys656Asn polymorphism and hypertension is still unclear and awaits further investigation.

To explore the interactive effects of obesity and the *LEPR* gene polymorphisms on hypertension, we analyzed the relation between polymorphisms (Lys109Arg, Gln223Arg and Lys656Asn) and hypertension by stratification analyses. We did not see a difference in the above *LEPR* polymorphisms on obesity. Masuo *et al.*^26^ reported that overweight obese subjects had significantly higher frequencies of the Arg223 (G) allele and the Arg223 (GG) homozygous allele of Gln223Arg and the Asn656 (C) allele of Lys656Asn compared with lean subjects in 129 young healthy normotensive men. However, a latest systematic review and analysis of primary data from the CoLaus study did not show an overall association between *LEPR* SNPs and overweight.^27^ In order to clearly define the relationships among *LEPR* polymorphism, obesity and hypertension, population stratification should be addressed in future genetic association studies.

Haplotype analysis is considered to be a powerful tool for studying the genetics of complex diseases. In our studies, two haplotypes with a frequency >1% were found, as previously described. There was linkage between Lys109Arg and Gln223Arg variations (*D*′=0.83, *r*^2^=0.48). Haplotype analyses showed that the G-A haplotype was a protective haplotype (*P*=0.012, OR=0.482, 95% CI 0.273–0.850), which was consistent with the findings of the association analyses between the Gln223Arg polymorphism and EH risk. Currently, little is known about the haplotype of the *LEPR* gene in EH population in the literature. Gu *et al.*^20^ did not conduct haplotype analysis in their report either. Therefore, similar studies using haplotype analysis in EH patients are still needed.

The consequences of the *LEPR* polymorphism on *LEPR* function are currently unclear. The three polymorphisms of the *LEPR* gene in our studies are all located in the intracellular domain of the receptor, indicating that they could potentially be functionally important for leptin signaling.^28^ Recent studies showed that the Gln223Arg polymorphism affected leptin signaling via STAT3.^29^ The Gln223Arg polymorphism in *LEPR* may also be in linkage disequilibrium with another functional polymorphism impairing signaling capacity of the LEPR.^30^ The Gln223Arg mutation led to an attenuation of the antiapoptotic effect of leptin. In the Zucker *fa/fa* rat model, a missense mutation^28^ in a highly conserved extracellular domain of the LEPR led to an elevated leptin level (hyperleptinemia);^31, 32^ whereas hyperleptinemia^33, 34^ is known to have a pathophysiological role in the development of hypertension and other cardiovascular diseases including coronary artery diseases.^35, 36^ In contrast, other reports suggested that the mutation of the *LEPR* gene may not directly influence the leptin level but could possibly advance the disease through inhibiting the biological effect of leptin.^37^

In summary, our studies clearly showed that the Gln223Arg polymorphisms in the *LEPR* gene are associated with EH risk in the Northern Han Chinese population. Interestingly, haplotype analyses suggested that the G-A haplotype was associated with the protection role of *LEPR* in EH development. Our studies suggested that the *LEPR* polymorphism had an important role in the susceptibility to EH in the Northern Han Chinese population. Meanwhile, experimental limitations are also present in our studies that could potentially affect our conclusions. For example, the age- and gender-matching between the case and control cohorts appear to be marginal as determined by their *P*-values, which could potentially influence our ability in detecting the associations between the SNPs and EH as well as other parameters. Our studies also did not cover the aspects of the functional research of *LEPR* gene polymorphisms, which might potentially overlook other gene variants that are strongly associated with EH. Future studies with larger sample size, more representative genetic backgrounds, multiple sample subgroups and functional research could potentially help further clarify the precise clinical roles of *LEPR* polymorphisms in the development of EH in different ethnic groups with distinct genetic backgrounds.


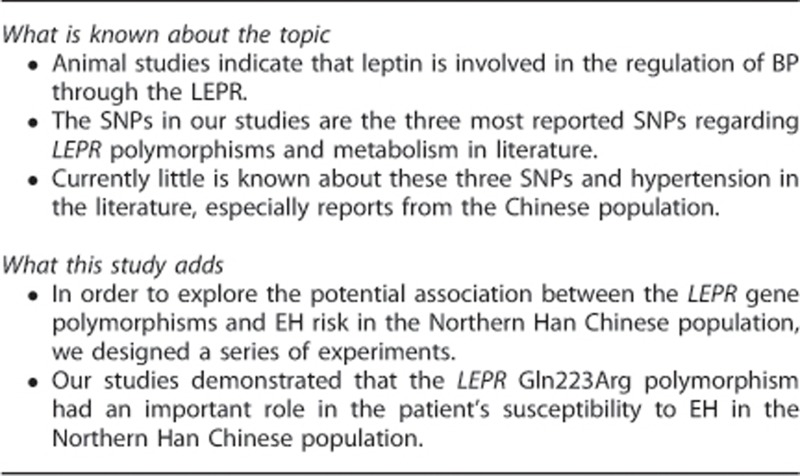


## Figures and Tables

**Table 1:** Clinical characteristics of normotensive and essential hypertensive participants

*Index*	*NT (491)*	*EH (823)*	P-*value*
Gender (M/F)	293/198	531/292	0.087
Age (years)	50.47±7.91	51.40±9.38	0.055
SBP (mm Hg)	107.43±8.46	161.86±18.31	**<0.001**
DBP (mm Hg)	69.22±6.90	102.73±14.10	**<0.001**
BMI (kg m^−2^)	25.08±3.37	26.96±3.50	**<0.001**
ALT (U l^−1^)	24.82±14.67	26.30±18.23	0.175
TG (mmol l^−1^)	1.63±1.53	1.88±1.53	**0.006**
TC (mmol l^−1^)	4.88±0.99	4.90±1.92	0.760
HDL-C (mmol l^−1^)	1.38±0.95	1.31±0.36	0.066
LDL-C (mmol l^−1^)	3.01±0.80	2.95±0.88	0.224
Glu (mmol l^−1^)	5.10±0.72	5.34±0.95	**<0.001**
BUN (mmol l^−1^)	5.70±3.86	5.89±2.88	0.439
Cr (μmol l^−1^)	77.71±14.92	79.11±19.21	0.221
HR (b.p.m.)	71.65±9.09	71.66±9.75	0.990
Smokers (*n*)	128	229	0.522
Drinkers (*n*)	75	248	**<0.001**

Abbreviations: ALT, alanine aminotransferase; BMI, body mass index; BUN, blood urea nitrogen; Cr, creatinine; DBP, diastolic blood pressure; EH, essential hypertensive patients; F, female; Glu, blood glucose; HDL-C, high-density lipoprotein cholesterol; HR, heart rate; LDL-C, low-density lipoprotein cholesterol; M, male; NS, not significant; NT, normotensive subjects; SBP, systolic blood pressure; TC, total cholesterol; TG, triglyceride.

All the quantitative data were presented as mean±s.d. *P*-values <0.05 are provided in bold to emphasize their significance.

**Table 2:** The frequencies of the *LEPR* gene Lys109Arg, Gln223Arg and Lys656Asn polymorphisms genotypes

	*Genotype (frequency, %)*			P-*value*	*Allele (frequency, %)*		P-*value*^a^
*Lys109Arg*							
	GG	AG	AA		G	A	
Case	547 (67.1)	246 (30.2)	22 (2.7)	0.601	1340 (82.2)	290 (17.8)	0.486
Control	323 (65.9)	149 (30.4)	18 (3.7)		795 (81.1)	185 (18.9)	

*Gln223Arg*							
Case	608 (75.2)	192 (23.8)	8 (1.0)	**0.041**	1408 (87.1)	208 (12.9)	0.189
Control	360 (73.5)	116 (23.7)	14 (2.9)		836 (85.3)	144 (14.7)	

*Lys656Asn*							
	GG	GC	CC		G	C	
Case	684 (86.6)	104 (13.2)	2 (0.3)	0.233	1472 (93.2)	108 (6.8)	0.098
Control	424 (89.8)	47 (10.0)	1 (0.2)		895 (94.8)	49 (5.2)	

a *P*-value of the comparison of allelic frequencies. *P*-value <0.05 is provided in bold to emphasize its significance.

**Table 3:** The genotype distributions and allele frequencies of the *LEPR* gene Lys109Arg, Gln223Arg and Lys656Asn polymorphisms in an obese and non-obese population

	*Genotype (frequency, %)*			P-*value*^a^	*Allele (frequency, %)*		P-*value*^b^
*Lys109Arg*							
	GG	AG	AA		G	A	
*Total*				0.875			0.893
Obese	545 (67.0)	243 (29.9)	26 (3.2)		1333 (81.9)	295 (18.1)	
Non-obese	325 (66.2)	152 (31.0)	14 (2.9)		802 (81.7)	180 (18.3)	

*Case*				0.415			0.908
Obese	395 (67.6)	171 (29.3)	18 (3.1)		961 (82.3)	207 (17.7)	
Non-obese	152 (65.8)	75 (32.5)	4 (1.7)		379 (82.0)	83 (18.0)	

*Control*				0.909			0.849
Obese	150 (65.2)	72 (31.3)	8 (3.5)		372 (80.9)	88 (19.1)	
Non-obese	173 (66.5)	77 (29.6)	10 (3.8)		423 (81.3)	97 (18.7)	

*Gln223Arg*							
*Total*				0.895			0.728
Obese	608 (75.0)	189 (23.3)	14 (1.7)		1405 (86.6)	217 (13.4)	
Non-obese	360 (73.9)	119 (24.4)	8 (1.6)		839 (86.1)	135 (13.9)	

*Case*				0.583			0.943
Obese	438 (75.4)	136 (23.4)	7 (1.2)		1012 (87.1)	150 (12.9)	
Non-obese	170 (74.9)	56 (24.7)	1 (0.4)		396 (87.2)	58 (12.8)	

*Control*				0.934			0.915
Obese	170 (73.9)	53 (23.0)	7 (3.0)		393 (85.4)	67 (14.6)	
Non-obese	190 (73.1)	63 (24.2)	7 (2.7)		443 (85.2)	77 (14.8)	

*Lys656Asn*							
	GG	GC	CC		G	C	
*Total*				0.407			0.658
Obese	693 (87.6)	95 (12.0)	3 (0.4)		1481 (93.6)	101 (6.4)	
Non-obese	415 (88.1)	56 (11.9)	0		886 (94.1)	56 (5.9)	

*Case*				0.440			0.515
Obese	497 (87.2)	71 (12.5)	2 (0.4)		1065 (93.4)	75 (6.6)	
Non-obese	187 (85.0)	33 (15.0)	0		407 (92.5)	33 (7.5)	

*Control*				0.464			0.369
Obese	196 (88.7)	24 (10.9)	1 (0.5)		416 (94.1)	26 (5.9)	
Non-obese	228 (90.8)	23 (9.2)	0		479 (95.4)	23 (4.6)	

a *P*-value of the comparison of the additive genetic model using the generalized linear model.

b *P*-value of the comparison of allelic frequencies.

**Table 4:** Odds ratios for each single-nucleotide polymorphism genotype associated with EH in the Northern Han Chinese population

*SNP*	*Model*	*Contrast*	*OR (95% CI)*	B	P-*value*
Lys109Arg	Allele comparison	G vs A	1.079 (0.867–1.342)	0.076	0.497
	Dominant genetic model	(GG+AG) vs AA	1.643 (0.823–3.279)	0.497	0.159
	Recessive genetic model	GG vs (AG+AA)	1.037 (0.805–1.337)	0.036	0.778
	Homozygote comparison	GG vs AA	1.667 (0.826–3.364)	0.511	0.154
	Additive genetic model	GG vs AG vs AA	1.081 (0.866–1.350)	0.078	0.490
Gln223Arg	Allele comparison	G vs A	1.165 (0.912–1.488)	0.153	0.221
	Dominant genetic model	(GG+AG) vs AA	3.697 (1.442–9.482)	1.308	**0.007**
	Recessive genetic model	GG vs (AG+AA)	1.076 (0.818–1.415)	0.073	0.599
	Homozygote comparison	GG vs AA	3.890 (1.501–10.077)	1.358	**0.005**
	Additive genetic model	GG vs AG vs AA	1.168 (0.913–1.495)	0.155	0.218
Lys656Asn	Allele comparison	C vs G	1.460 (1.011–2.108)	0.378	**0.044**
	Dominant genetic model	(CC+GC) vs GG	1.515 (1.032–2.225)	0.415	**0.034**
	Recessive genetic model	CC vs (GC+ GG)	0.920 (0.080–10.565)	−0.084	0.946
	Homozygote comparison	CC vs GG	0.966 (0.084–11.128)	−0.034	0.978
	Additive genetic model	CC vs GC vs GG	1.479 (1.017–2.152)	0.392	**0.041**

Abbreviations: B, coefficients; CI, confidence interval; OR, odds ratio; SNP, single-nucleotide polymorphism.

ORs adjusted for age, gender, body mass index, total cholesterol, high-density lipoprotein cholesterol, low-density lipoprotein cholesterol, triglyceride level, glucose level, smoking habits and drinking habits. *P*-values <0.05 are provided in bold to emphasize their significance.

**Table 5:** Haplotype analyses of the *LEPR* gene polymorphisms in hypertension and control subjects

		*Haplotype frequency*					
*Lys109Arg*	*Gln223Arg*	*Cases*	*Controls*	*HS test* P*-value*^a^	*OR (95% CI)*^a^	P*-value*^b^	*OR (95% CI)*^b^
G	G	0.808	0.784	0.132	1.162 (0.956–1.413)	—	—
A	A	0.114	0.120	0.647	0.944 (0.738–1.207)	0.519	1.085 (0.847–1.390)
A	G	0.065	0.069	0.660	0.931 (0.679–1.278)	0.550	1.102 (0.802–1.514)
G	A	0.014	0.028	**0.012**	0.482 (0.273–0.850)	**0.010**	0.474 (0.269–0.837)

Abbreviations: CI, confidence interval; HS test, haplotype-specific testing; OR, odds ratio.

All haplotypes with frequency >1% detected in the haplotype analyses are shown in the table.

a *P*-values and OR values derived from comparing of a specific haplotype with the other three.

b *P*-values and OR values derived from comparing each haplotype with the baseline haplotype (G-G). *P*-values <0.05 are provided in bold to emphasize their significance.
